# A Ranking-Based Meta-Analysis Reveals Let-7 Family as a Meta-Signature for Grade Classification in Breast Cancer

**DOI:** 10.1371/journal.pone.0126837

**Published:** 2015-05-15

**Authors:** Yasemin Oztemur, Tufan Bekmez, Alp Aydos, Isik G. Yulug, Betul Bozkurt, Bala Gur Dedeoglu

**Affiliations:** 1 Ankara University, Biotechnology Institute, Ankara, Turkey; 2 Gazi University, Faculty of Dentistry, Ankara, Turkey; 3 Bilkent University, Molecular Biology and Genetics Department, Ankara, Turkey; 4 Ankara Numune Training and Research Hospital, Department of General Surgery, Ankara, Turkey; Cankiri Karatekin University, TURKEY

## Abstract

Breast cancer is one of the most important causes of cancer-related deaths worldwide in women. In addition to gene expression studies, the progressing work in the miRNA area including miRNA microarray studies, brings new aspects to the research on the cancer development and progression. Microarray technology has been widely used to find new biomarkers in research and many transcriptomic microarray studies are available in public databases. In this study, the breast cancer miRNA and mRNA microarray studies were collected according to the availability of their data and clinical information, and combined by a newly developed ranking-based meta-analysis approach to find out candidate miRNA biomarkers (meta-miRNAs) that classify breast cancers according to their grades and explain the relation between miRNAs and mRNAs. This approach provided meta-miRNAs specific to breast cancer grades, pointing out let-7 family members as grade classifiers. The qRT-PCR studies performed with independent breast tumors confirmed the potential biomarker role of let-7 family members (meta-miRNAs). The concordance between the meta-mRNAs and miRNA target genes specific to tumor grade (common genes) supported the idea of mRNAs as miRNA targets. The pathway analysis results showed that most of the let-7 family miRNA targets, and also common genes, were significantly taking part in cancer-related pathways. The qRT-PCR studies, together with bioinformatic analyses, confirmed the results of meta-analysis approach, which is dynamic and allows combining datasets from different platforms.

## Introduction

Breast cancer (BC) is the second leading cause of cancer-related deaths worldwide in women, after lung cancer. Despite advances in early detection and treatment, the estimated number of deaths in 2014 due to BC is about 40,000. The chance of a woman having invasive breast cancer during her lifetime is about 1 in 8. The chance of dying from breast cancer is about 1 in 36 [1]. BC is a complex and heterogeneous disease with varied molecular features, tumor characteristics, expression patterns and response to therapy. It has been studied in detail at the molecular level. Although some genes were found to be responsible for the development and progression of the disease, most of the molecular mechanisms underlying its progression remain poorly understood. This has led to a significant interest in the quest for novel predictive markers for BC [2,3].

The clinical management of breast cancer depends on the availability of robust clinical and pathological prognostic factors. One of the best-established prognostic factors for BC is histological grade, which is based on the degree of differentiation of the tumor tissue. Recent microarray-based expression profiling studies have provided evidence that tumors of different grades show distinct genomic, transcriptomic and immunohistochemical profiles [4].

MicroRNAs (miRNAs) are small non-coding RNA molecules, which negatively regulate gene expression at the post-transcriptional level [5]. Following the discovery of the first miRNA in the roundworm *Caenorhabditis elegans*, these short regulatory RNAs have been found to be an abundant class of RNAs in plants, animals, and DNA viruses. miRNAs induce mRNA degradation (some studies indicate that destabilization of target mRNAs is the predominant reason for reduced protein output), translational repression, or both depending upon the complementarity of the miRNA to its mRNA target [6–8]. miRNAs play a crucial role in normal biological processes including development, cell proliferation, differentiation, and apoptosis [9]. Future work on miRNAs and miRNA microarray studies will elucidate new areas of cancer development and progression. Intensifying research in miRNA studies, using high throughput techniques including microarrays has resulted in the identification and confirmation of aberrant miRNA expression in a number of human diseases including BC [3,5].

The let-7 family of miRNAs was initially discovered as important developmental molecules in *C*. *elegans*. It is known that mature let-7 family members are highly conserved across animal species and that these miRNAs are key regulators of cell proliferation and differentiation. Let-7 family members are often reported as tumor suppressors in humans. Altered let-7 family expression is likely to contribute to cancer formation and also progression. Accordingly, these miRNAs may have the potential to be used as novel predictive markers in cancer [10,11].

Microarray technology has been widely used in research to find new biomarkers in the past decade. Many transcriptomic microarrays have been generated and made available in public databases such as the Gene Expression Omnibus (GEO)[12] from NCBI (http://www.ncbi.nlm.nih.gov/geo/) and Array Express[13] from EBI (http://www.ebi.ac.uk/arrayexpress/) [14,15].

Meta-analysis may briefly defined as combining independent studies using various methods to increase the statistical power by increasing the number of samples analyzed [16,17]. Meta-analysis approaches have the potential to enable more comprehensive evaluation of the existing differential miRNA expression data and can therefore provide miRNA and miRNA-target gene sets with a high diagnostic value. In addition to ongoing discovery of new miRNAs and inconsistent annotation, platform variability, various methods for data processing, and analysis are the limitations for combining independent datasets [17].

To overcome these limitations, we developed a ranking-based meta-analysis approach. It is known that ranking-based meta-analysis approaches are successful in eliminating the differences that originate from platform variability [18]. The method enabled us to combine three independent miRNA microarray studies performed with three different platforms and two different mRNA microarray studies performed with the same platform. Herein we provide miRNA and mRNA lists that are discriminative specifically for breast cancer grades, which are invariably expressed across independent experiments. In the miRNA list, eight of the let-7 family members were found to be top-ranking miRNAs with a discriminative power for breast cancer grades and were validated by qRT-PCR. Consequently to the best of our knowledge this is the first meta-analysis study that combines and correlates miRNA and mRNA microarray studies conducted in breast cancer.

## Material and Methods

**Fig 1:**
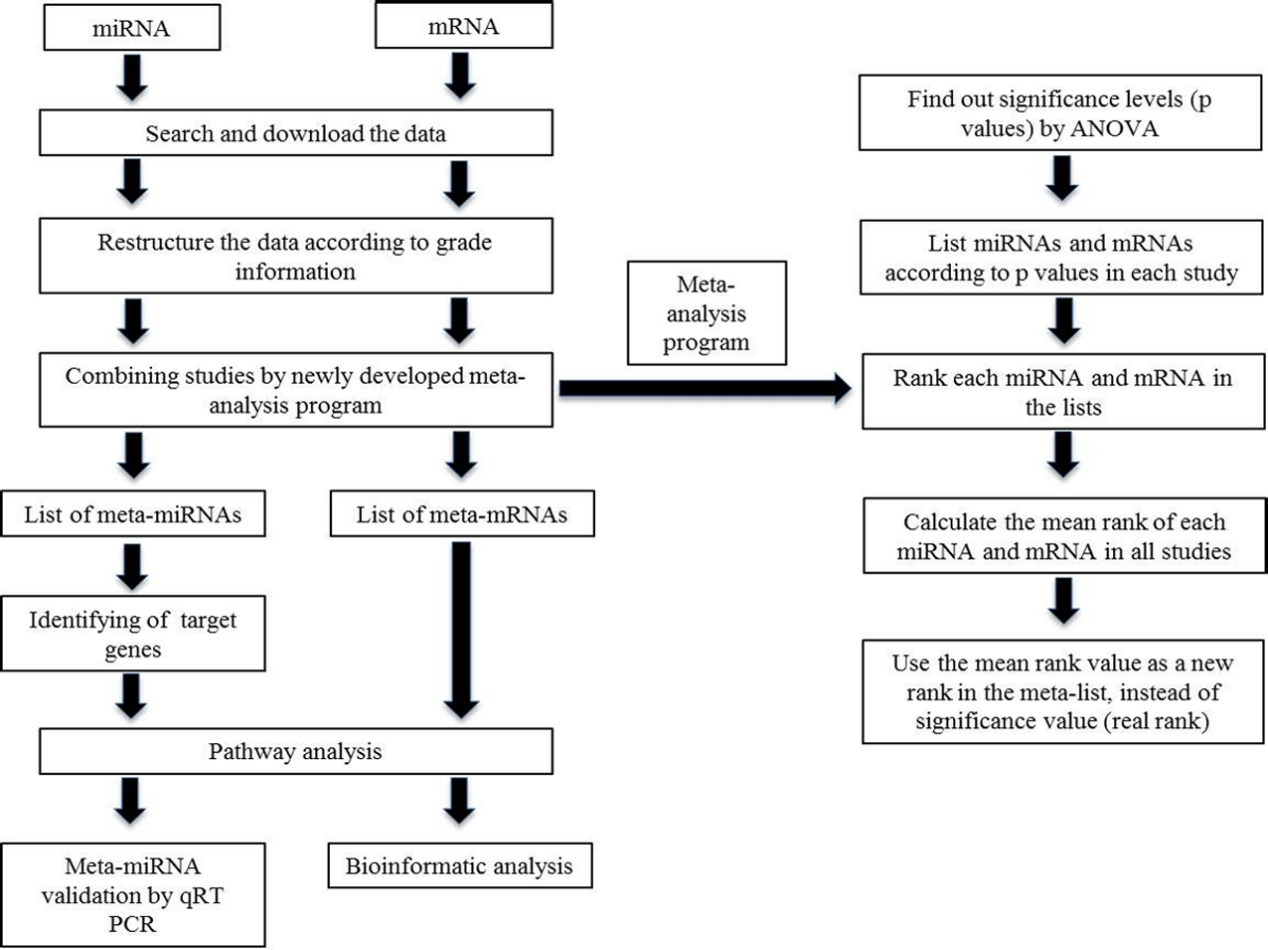
General study and meta-analysis scheme. The workflow is represented by boxes and arrows.

We explained the relation between miRNAs and mRNAs in addition to the identification of miRNA signatures in this study. Accordingly the study steps were designed for both miRNA and mRNA together (Fig 1).

### Selection and restructuring of datasets

miRNA and mRNA microarray studies related to breast tumors were searched in two microarray databases, Gene Expression Omnibus [12] (GEO, www.ncbi.nlm.nih.gov/geo/) and ArrayExpress [13] (www.ebi.ac.uk/arrayexpress) using the search terms “miRNA” or “mRNA”, “microarray” and “breast cancer” together. With these keywords we found 96 studies in which 52 of the microarray studies were performed with cell lines and 4 of them were formalin-fixed, paraffin-embedded (FFPE) studies. The datasets that were generated by using cell lines and FFPE tissue samples were excluded. The rest of the datasets were evaluated according to the presence of their data and grade information. Only studies with miRNA and mRNA microarray expression data conducted in human breast cancer tissues that had clinical information regarding grade status were included for further analysis.

Three miRNA datasets (GSE7842 [19], GSE15885 [20] and GSE22216 [21]) and 2 mRNA datasets (GSE17907 [22] and E-MTAB-1006 [23]) were selected and downloaded as a “series matrix” file and “processed data” file from GEO and ArrayExpress respectively. Each dataset was then annotated and grade information files were restructured for standardization.

### miRNA and mRNA datasets

Three breast cancer miRNA microarray studies and 2 breast cancer mRNA microarray studies were retrieved from GEO and ArrayExpress (Table 1). All 5 datasets were taken from full-text research articles published between 2007 and 2011.

**Table 1:** The characteristics of the datasets used for meta-analysis.

miRNA studies	Platform	Grade 1	Grade 2	Grade 3
GSE7842	Luminex Bead-based microRNA profiling platform version 3	20	34	39
GSE15885	Exiqon Homo sapiens 0.4K miChip v8	5	7	17
GSE22216	Illumina Human v1 MicroRNA expression beadchip	42	81	63
Total		67	122	119
**mRNA studies**				
E-MTAB-1006	Affymetrix Human Genome U133 Plus 2.0 Array	18	34	44
GSE17907	Affymetrix Human Genome U133 Plus 2.0 Array	3	10	34
Total		21	44	78
**in silico test study**				
GSE22219	Illumina human Ref-8 v1.0 expression beadchip	41	87	62

GSE7842 miRNA data from Blenkiron et al. [19] were generated using the Luminex Bead-based microRNA profiling platform version 3. This dataset contained 93 primary human breast tumors (20 samples were Grade 1, 34 samples Grade 2 and 39 samples Grade 3).

GSE15885 miRNA data from Lowery et al. [20] were generated using the Exiqon Homo sapiens 0.4K miChip v8. This dataset contained 29 early stage breast cancer specimens (5 samples were Grade 1, 7 samples Grade 2 and 17 samples Grade 3).

The last miRNA data GSE22216 from Buffa et al. [21] were generated using the Illumina Human v1 MicroRNA expression beadchip. This dataset contained 210 early primary breast cancer samples (42 samples were Grade 1, 81 samples Grade 2, 63 samples Grade 3 and 24 of an unknown grade).

GSE17907 [22] and E-MTAB-1006 [23] mRNA datasets were both generated using the Affymetrix Human Genome U133 Plus 2.0 Array. GSE17907 contained 55 breast cancer samples (3 samples were Grade 1, 10 samples Grade 2, 34 samples Grade 3 and 8 of an unknown grade) while E-MTAB-1006 contained 96 breast cancer samples (18 samples were Grade 1, 34 samples Grade 2 and 44 samples Grade 3).

The independent mRNA microarray test data GSE22219 from Buffa et al. [21] that was used for in silico analysis were generated using the Illumina human Ref-8 v1.0 expression beadchip. This dataset contained 190 primary human breast tumors (41 samples were Grade 1, 87 samples Grade 2 and 62 samples Grade 3).

### Meta-analysis

The limitations of meta-analysis of miRNA studies are inconsistent annotation, platform variability, various methods for data processing, and analysis resulting from the usage of different platforms. To eliminate such limitations for combining independent datasets an ANOVA-dependent ranking-based meta-analysis program was developed by using MATLAB. ANOVA tests were performed to find the significance levels (p values) among samples with grade I, II and III. For each study, the miRNA and mRNA lists were generated considering their p values, and miRNAs and mRNAs were noted according to their ranks in the list. For each miRNA or mRNA, the mean of its rank in all studies was calculated and this value became its *real rank* in the meta-list, which was used instead of the significance value (Fig 1).

### Pathway enrichment analysis

**Fig 2:**
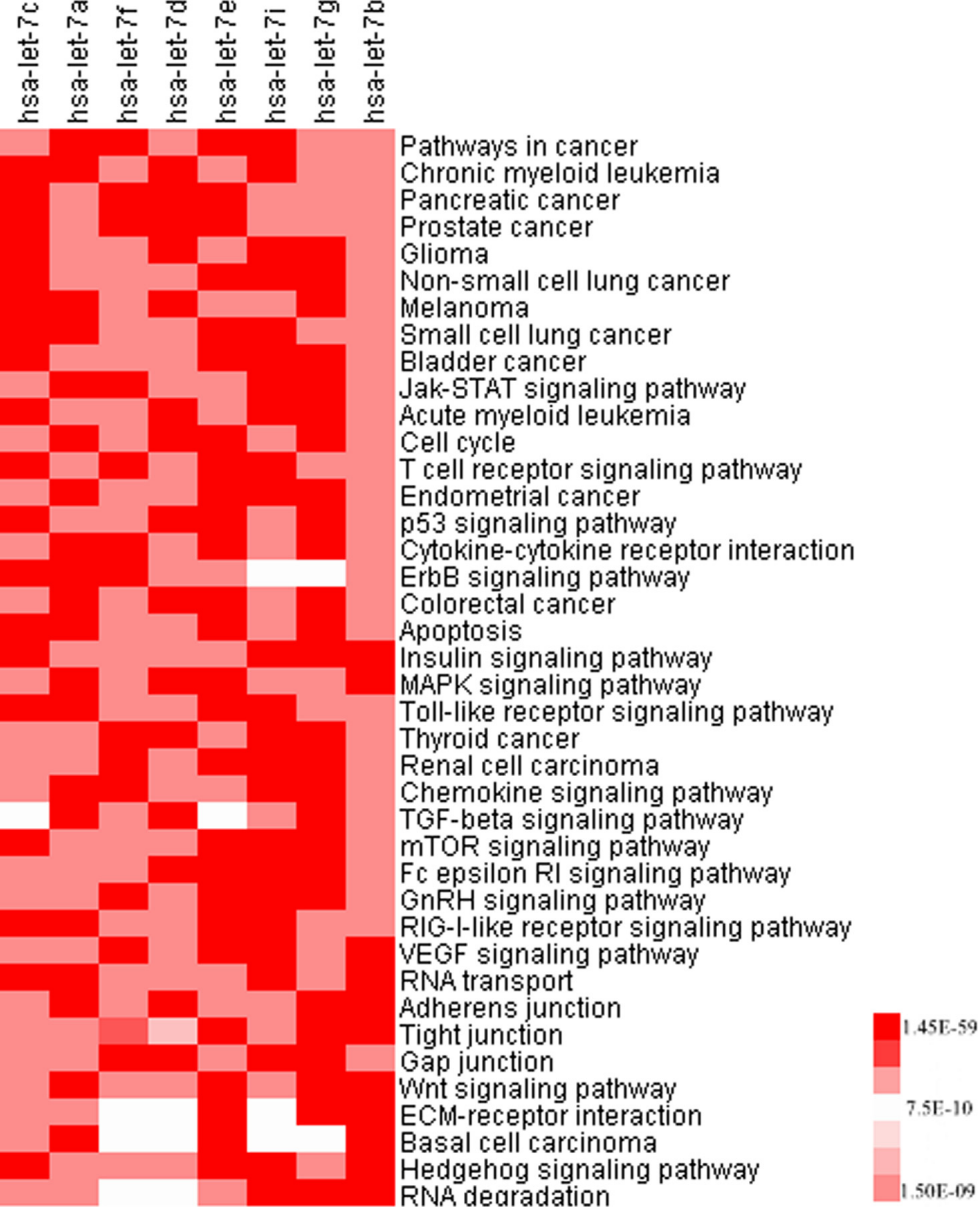
Pathway enrichment of the grade-specific let-7 family targets. Validated targets of the let-7 miRNAs and KEGG pathway results were used to construct the heatmap. The intensity of color represents the adjusted p value (p≤0.0091).

The WebGestalt [24] (http://bioinfo.vanderbilt.edu/webgestalt/) pathway analysis tool was used for enrichment analysis of the targets of the let-7 family members and the common targets of the meta-miRNAs and meta-mRNAs. The enrichment results of the let-7 family target genes were visualized by a heatmap (Fig 2). The KEGG pathway enrichment results of the validated target genes and FDR corrected p values were used as input for the Cluster 3.0 [25] and TreeView [26] programs.

### Validation of meta-miRNAs by Real-Time quantitative RT-PCR (qRT-PCR)

#### Breast tumor samples

Primary breast tumor samples were obtained from the patients during the surgery, instantly snap-frozen in liquid nitrogen, and stored at -80°C until RNA extraction. The total number of samples was 21. Four of the breast tumor samples were Grade 1, 11 were Grade 2 and 6 were Grade 3. This study and the use of the tissue material in this project were approved by the Research Ethics Committee of Ankara University School of Medicine. The written consent was obtained from the patients in accordance with the Helsinki Declaration.

#### RNA extraction and cDNA synthesis

The RNA isolation protocol was performed according to the previous study [27]. Briefly, the frozen breast tumor samples were put into Trizol reagent (AppliChem, Darmstadt, Germany), disrupted with a homogenizer, and total RNA was isolated according to the manufacturer’s instructions. Genomic DNA contamination was removed by on-column DNase treatment (Macharel Nagel, Duren, Germany). The concentration of the isolated RNA and ratio of absorbance at 260 nm to 280 nm were measured with the NanoDrop ND-1000 spectrophotometer (NanoDrop Technologies, Montchanin, DE, USA).

First-strand cDNA was synthesized from 1 μg total RNA with the miScript II RT Kit according to the manufacturer’s instructions (Qiagen, Hilden, Germany). In a reverse-transcription reaction, mature miRNAs were polyadenylated by poly(A) polymerase and subsequently converted into cDNA by reverse transcriptase with oligo-dT priming. The cDNA was then used for qRT-PCR profiling of mature miRNA expression. The cDNA was diluted at a ratio of 1:10 before being used as a PCR template and stored at -20°C until further use.

#### qRT-PCR

qRT-PCR analysis was performed on the Roche LightCycler 480 by using specific miRNA primers and the miScript SYBR Green PCR Kit (Qiagen, Hilden, Germany). The amplification mixtures contained 1.0 μl 1:10 diluted cDNA, 5.0 μl SYBR Green PCR Master Mix, 1.0 μl miScript Universal Primer (reverse primer for all miRNAs), 1.0 μl miScript Primer Assay (miRNA specific forward primer) and 2 μl RNase-free water in a total volume of 10 μl. Cycling conditions were as follows: 95°C for 15 min for initial activation and then 40 cycles of 94°C for 15 s, 55°C for 30 s, and 70°C for 30 s during amplification. The ΔΔCt method was used for the analysis of qRT-PCR results and RNU6-2 was used as a normalization factor.

### Prediction of miRNA targets and their comparison with meta-mRNAs

The validated targets of the grade-specific meta-miRNAs were identified bioinformatically by using miRWalk [28] (http://www.umm.uni-heidelberg.de/apps/zmf/mirwalk/index.html). miRWalk is a comprehensive database that involves not only in silico predicted but also experimentally validated targets. To compare the meta-miRNA targets with meta-mRNAs, common genes were identified by using Venny [29] (http://bioinfogp.cnb.csic.es/tools/venny/)

### In silico analysis of common genes

The independent mRNA microarray data GSE22219 [21] that contains the grade information was reanalyzed by BRB-ArrayTools [30] to analyze the prediction power of common genes between validated meta-miRNA targets and meta-mRNAs. Hierarchical clustering of common genes and tumor samples according to their grade information was performed by using average linkage as a clustering method.

The interaction network between grade predictive let-7 family members and their potential target genes validated by in silico analysis of common genes was visualized by using Cytoscape 3.2.0 network analysis and visualization platform [31].

## Results

### miRNA specific datasets and meta-analysis

In this study to combine independent microarray studies coming from different platforms ranking-based meta-analysis approach was developed. After evaluation processes we picked 3 miRNA microarray studies conducted in breast cancer tumors, which have clinical information regarding grade status and available microarray data. Consequently we could apply the meta-analysis approach to 308 samples from 3 different miRNA microarray studies and 67 samples were Grade 1, 122 Grade 2 and 119 Grade 3 (Table 1).

The number of probes in each data group was 359 for GSE7842 [19], 396 for GSE15885 [20] and 735 for GSE22216 [21]. Accordingly the total number of miRNAs included in the study was 551. The meta-analysis program uses the average values to calculate the *real rank* of a miRNA, decreasing the number of miRNAs to 194 at the end of the meta-analysis (S1 Table).

Although the rank of any given miRNA varied from low to high in the individual datasets, this was stabilized with the *real rank* at the end of the meta-analysis. In the case of let-7c, the rank was 11 in GSE7842 [19], 4 in GSE15885 [20] and 12 in GSE22216 [21] according to the ANOVA results but it took 1^st^ position as its *real rank* in the meta-list with a mean rank of 9 (Table 2).

**Table 2:** Top 20 meta-miRNAs and their ranking values.

miRNA name	the mean of rank	real rank
hsa-let-7c*	9	1
hsa-let-7a*	12	2
hsa-let-7f*	14.5	3
hsa-let-7d*	15.5	4
hsa-let-7e*	19	5
hsa-let-7i*	23.7	6
hsa-miR-30a-3p	26	7
hsa-let-7g*	31.3	8
hsa-miR-331	53.5	9
hsa-miR-199b	55.5	10
hsa-let-7b*	71	11
hsa-miR-199a	71.5	12
hsa-miR-30a-5p	73	13
hsa-miR-509	75	14
hsa-miR-7	85.5	15
hsa-miR-320	86.5	16
hsa-miR-33	90	17
hsa-miR-28	91.5	18
hsa-miR-99a	91.5	19
hsa-miR-122a	92.5	20

The mean rank indicates the average of rank values in each study for a given miRNA and the real rank is the rank of a given miRNA in the meta-list generated by the meta-analysis approach.

* indicates the let-7 family members in the meta-list that were chosen for further analysis.

### Let-7 family members are enriched in meta-miRNA list

As provided in S1 Table, 194 miRNAs were listed according to the mean of their ranks and they were ordered from 1 to 194 as their *real rank* scores. Since the *real rank* in the meta-list was used instead of the significance value we focused on the first 20 meta-miRNAs listed in Table 2. The abundance of let-7 family members in the top 20 meta-miRNA list was remarkable. Eight of the top 20 was found to be consisting of let-7 family members. The first 6 miRNAs in the meta-list were let-7c, let-7a, let-7f, let-7d, let-7e, let-7i and let-7g was the 8^th^and let-7b was the 11^th^ miRNAs (Table 2). Additionally hsa-miR-7, hsa-miR-99a, hsa-miR-320, hsa-miR-30a-3p and hsa-miR-30a-5p that were previously associated with breast cancer were also in the meta-list [32–37]. The amount of cancer related miRNAs in the first top 20 meta-list might be the indication of the robustness of the miRNAs reliability of them to be real grade predictors.

### Let-7 family targets are associated with cancer specific pathways

To further characterize the relation of let-7 family members with the grade status of breast tumors, the validated targets of the 8 grade-specific meta-miRNAs were identified by miRwalk [28]. Among all 8 let-7 family members let-7b was found to have the highest number of defined targets with 663 target genes and let-7i was found to have 269 validated target genes, which was the lowest target number. On the other hand the number of targets was 299 for hsa-let-7c, 308 for hsa-let-7a, 283 for hsa-let-7f and hsa-let-7d, 301 for hsa-let-7e and 273 targets for hsa-let-7g.

The target gene lists that were obtained for each miRNA were used for pathway enrichment analysis that was performed by WebGestalt [24]. The KEGG pathway enrichment results of the validated target genes were extracted and visualized by a heatmap, which was generated by using FDR corrected p values of enrichment analysis. The results showed that most of the target genes were taking part in cancer-related pathways such as the cell cycle, apoptosis and signaling pathways including MAPK, p53, ErbB, Wnt, Jak-STAT and TGF-beta (Fig 2; p<0.0091). It was also interesting to mention that none of the targets of miRNAs missed any given pathway and all of them shared each and every pathway given in Fig 2 within a range of significance between 10^–3^ and 10^–59^.

### qRT-PCR experiments validated the grade predictive ability of meta-miRNAs in independent tumor samples

**Fig 3:**
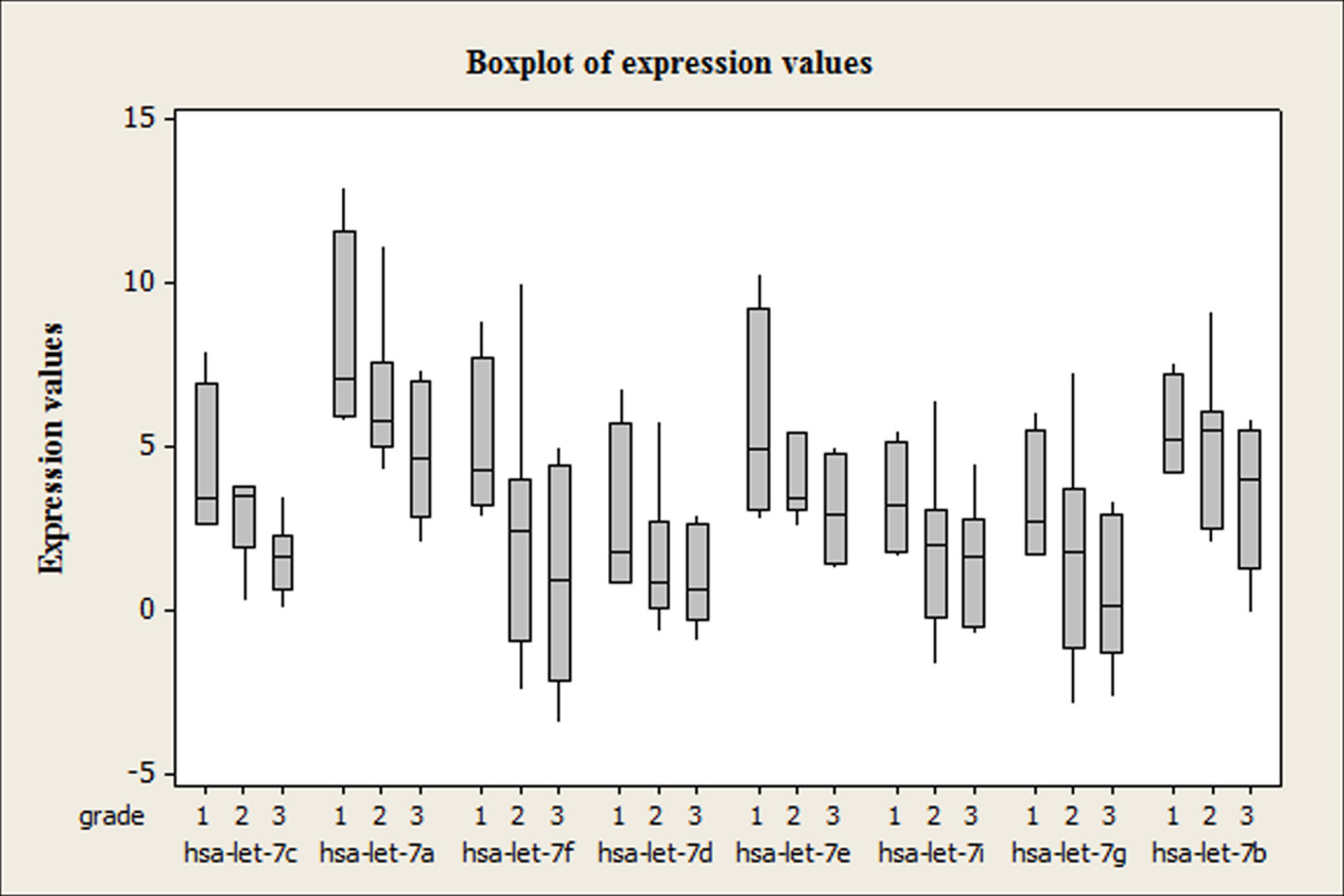
Boxplot of let-7 family expression levels. A consistent decrease in the expression levels of let-7 family members is observed from grade 1 to grade 3 tumors (n = 21).

Expression profiles of the eight let-7 family members were tested in independent breast tumors to validate meta-analysis results by qRT-PCR (n = 21). The expression range of each miRNA in each grade (Grade1, Grade 2 and Grade 3) was shown in terms of normalized Ct values according to a normalization factor RNU6 as box-whisker-plots in Fig 3. Concordant with the meta-analysis results the expression levels of all *let-7* family members were found to be consistently downregulated with increasing tumor grade. Let-7d, let-7i and let7g were observed to be similar in terms of expression profiles throughout the tumor samples representing each tumor grade. Since a very wide range of distribution in the expression values of most of the miRNAs in Grade 2 tumor samples were observed, none of the let-7 family members were found to be discriminative between Grade 1 and 2 and Grade 2 and 3 tumors. On the other hand the changes in the expression levels of hsa-let-7a, hsa-let-7c, hsa-let-7e in Grade 1 and Grade 3 tumors were found to be statistically significant (p≤0.05) (Fig 3).

### In silico integrated analysis of meta-miRNA-targets and meta-mRNAs resulted common mRNAs specific to grade status

To validate the reliability of let-7 family members in grade status discrimination, an integrated analysis was designed in which the targets of the let-7 family members were compared with the meta-mRNAs that are supposed to be grade predictors. For this integrated analysis the meta-analysis approach that was applied to miRNA datasets was also applied to the 2 mRNA microarray studies conducted in breast cancer (Table 1) and meta-mRNAs that could classify breast cancer tumors according to their grade status were ranked. What we expected was to find out common genes between grade specific miRNA-targets and grade specific meta-mRNAs. That is because if we could find any mRNAs that are discriminative between grade statuses, they had to share some genes with the targets of grade predictive-miRNAs.

**Fig 4:**
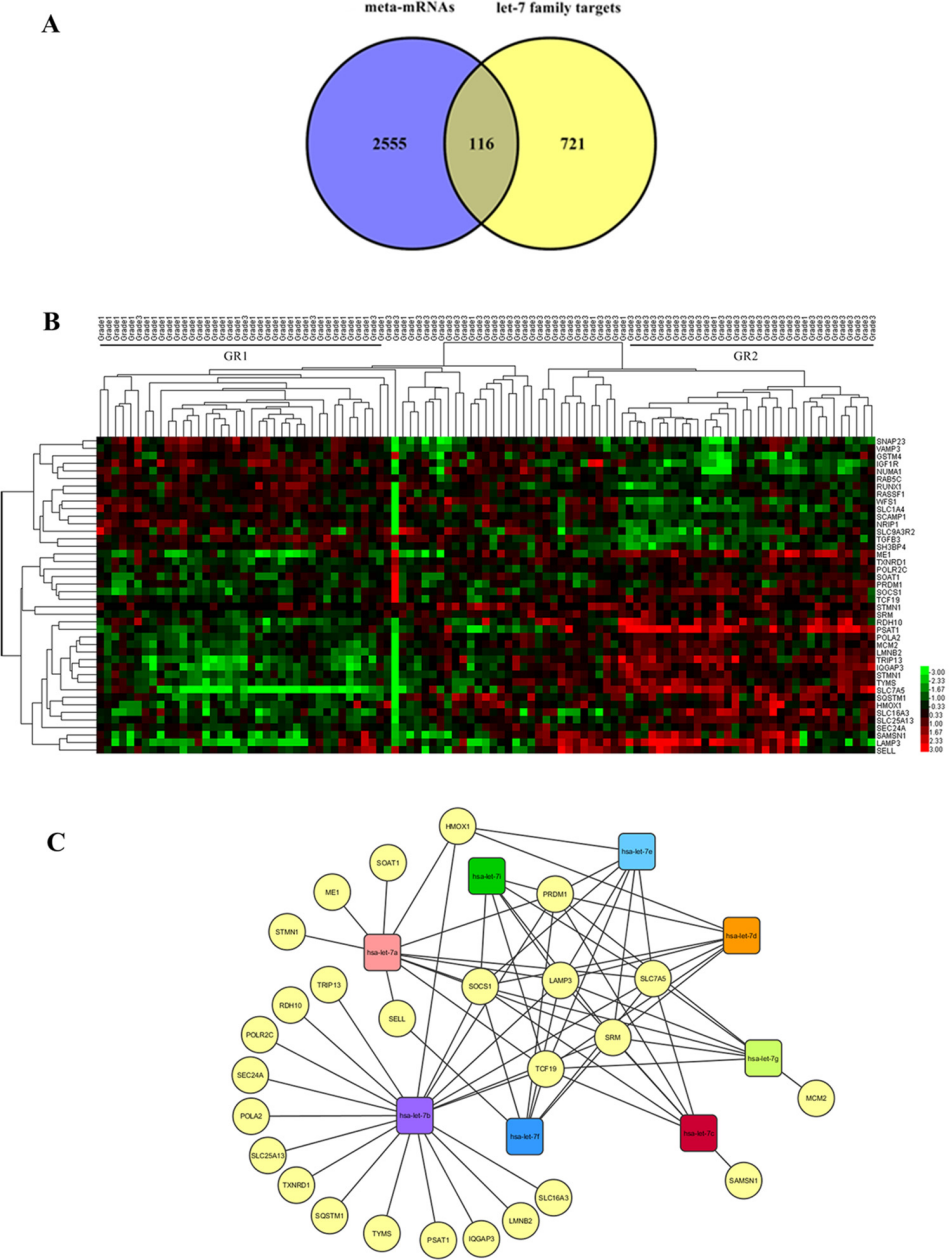
In silico analysis of the common genes between validated meta-miRNA targets and meta-mRNAs. (A) The intersection of meta-mRNAs and let-7-target genes. 116 of the meta-miRNA targets are in common with the meta-mRNAs. (B) Heatmap of the expression of common genes between grade-specific let 7 targets and mRNAs for the GSE22219 dataset. Hierarchical clustering of common genes and grade 1 and grade 3 tumor samples was performed by using average linkage as the clustering method. Red and green colors represent upregulation and downregulation, respectively (p≤0.05). (C) The interaction network between grade predictive let-7 family members and their potential target genes validated by in silico analysis. Target genes are represented by yellow and circular nodes while miRNAs are represented by squares.

The intersection of meta-mRNAs (top 20% of 13355 mRNAs) and let-7-target genes (837 targets without repetition) was found by Venny analysis and the results showed that they shared 116 common genes (Fig 4A, S2 Table). The pathway enrichment analysis results showed that most of the genes common to meta-miRNA targets and meta-mRNAs took part in important pathways such as cancer specific pathways, the cell cycle, TGF-beta signaling pathway, apoptosis, MAPK, mTOR and VEGF signaling pathways (p≤ 0.0205).

To further validate the prediction power of the 116 common genes an independent microarray data from GSE22219 [21] were reanalyzed and expression results were visualized by a heatmap. Primary breast tumors were hierarchically clustered into two major groups based on target gene expression profiles. This in silico validation showed that 41 of the 116 genes were able to significantly cluster Grade 1 and Grade 3 tumors. Grade 1 tumors were significantly located in the first group (GR1) while the samples in the second group (GR2) were mostly Grade 3 tumors (Fig 4B; Mann-Whitney; W = 1642, p = 0.0000). As expected, some of the target mRNA expression levels were observed to be increased with increasing grade status, unlike the miRNA expression levels. The genes that were upregulated in grade 3 tumors may be thought to be the direct targets of let-7 family members because of their inverse correlation and include ME1, TXNRD1, POLR2C, SOAT1, PRDM1, SOCS1, TCF19, STMN1, SRM, RDH10, PSAT1, POLA2, MCM2, LMNB2, TRIP13, IQGAP3, TYMS, SLC7A5, SQSTM1, HMOX1, SLC16A3, SLC25A13, SEC24A, SAMSN1, LAMP3 and SELL (Fig 4B). To visualize the interactions of each gene with the miRNAs cytoscape network analysis and visualization platform was used. The network analysis indicated that half of the 26 potential target genes were unique to let-7b and this miRNA shared 8 targets with the other let-7 family members. Additionally PRDM1, SOCS1, TCF19, SRM, SLC7A5 and LAMP were located as the genes targeted by all of the grade predictive let-7 family members (Fig 4C). The KEGG pathway analysis results indicated that among 26 genes some of them were found to have roles in cancer related pathways like cell cycle (MCM2), Jak-STAT (SOCS1), MAPK (STMN1), PPAR signaling pathways (ME1).

## Discussion

Meta-analysis is a procedure for statistically combining the results of many different studies. The data obtained by meta-analysis is more generalized and stable since the information is not specific to a case but the combination of many different studies. The common problem of various meta-analysis approaches is the technical differences resulting from the usage of different platforms. This necessitates the development of new methodologies with the potential to eliminate the differences resulting from the different probe design, normalization and preprocessing methods specific to each platform. We combined independent miRNA microarray datasets to identify miRNA signatures that could classify the breast cancer samples according to their histological grade in this study. An ANOVA-dependent ranking-based meta-analysis approach that makes identification of novel candidate miRNAs possible with the potential to eliminate differences emerging from microarray platform variability was developed in MATLAB for this purpose. The code is so dynamic that it allows the addition of newly generated data for meta-analysis. In a ranking-based meta-analysis study, Phan et al. compared their Rank Average Meta-Analysis with 5 other meta-analysis approaches (mDEDs, rank products, Choi, Wang and naive methods) and found that combining microarray datasets by averaging ranks effectively increases the sample size while enabling robust analysis of heterogeneous data.[18] Another ranking-based methodology was proposed by Kolde et al. as a novel rank aggregation method that is free and robust as regards outliers, noise and errors [38]. Ranking-based algorithms are therefore suggested to be useful and general solutions for the integration of variable datasets.

The method enabled us to combine three independent miRNA microarray studies performed with three different platforms and two different mRNA microarray studies performed with the same platform. Since miRNA microarray studies are mostly concentrated on the comparison of tumor and normal tissues, the pathological information (including the grade information) was not available, which limited the number of studies we used for meta-analysis.

The meta-analysis study was performed with the tumor samples obtained from independent studies in which the clinical information of the tumors was available. In this study we concentrated on meta-miRNAs that are predictive for histological grade status, one of the important prognostic factors in breast cancer. In the meta-miRNA list obtained from the meta-analysis, 8 of the first 20 miRNAs were found to be let-7 family members; let-7c, let-7a, let-7f, let-7d, let-7e, let-7i, let-7g and let-7b (Table 2). The enrichment of let-7 family members in the meta-list led us to focus on the let-7 family and to further investigate their role in grade prediction. We started with the experimental confirmation of in silico predictions by qRT-PCR. The results of the qRT-PCR performed with independent breast cancer samples pointed out that the aforementioned eight let-7 family members were differentially expressed between grade 1 and grade 3 tumors and decreased miRNA expression levels were observed with increasing tumor grade. Among these miRNAs, the decrease in the expression of let-7a, let-7c and let-7e was found to be statistically significant (Fig 3; n = 21, p≤0.05). Similar to our results, in a study of Blenkiron et al., decreased expression levels of these three miRNAs with increasing tumor grade was shown in a heatmap. Although their focus was not specifically grade status and any let-7 family expression profiles they gave a list of miRNAs differentially expressed among tumor grades and the data was extracted from a heatmap that was given in the paper [19]. Grading shows the differentiation status of the tumors. Grade 1 tumor cells appear close to normal and are differentiated while Grade 3 tumors are poorly differentiated. What we observed in the qRT-PCR experiments was a consistent decrease in the expression levels of miRNAs from differentiated to poorly differentiated tumors (Fig 3). These findings were supported by a study in which general miRNA expression levels and differentiation status of the tumors were found to be inversely correlated [39]. These findings were also consistent with the report that mouse embryonic stem cells lacking Dicer, an important enzyme for miRNA maturation, fail to differentiate normally [40]. By reanalyzing the let-7 family members of a very recent study (GSE60725), it was shown that the same family members were downregulated in grade 3 tumors compared to grade 2 tumors. Better differentiation of the tumors was therefore positively correlated with higher miRNA expression levels. Rothe et al. reported that hsa-miR-93, hsa-miR-423, hsa-miR-25, hsa-miR-106b and hsa-miR-345 that are in top 50 in our meta-miRNA list were biomarker candidates for grade prediction between grade 1 and grade 3 tumors.[41] In addition to the let-7 family members, the qRT-PCR results confirmed that miR-30a-3p and miR-30a-5p, which were listed among the top 20 meta-miRNAs, were significantly discriminative between Grade 1 and Grade 2 tumors (p<0.01). The expression levels were found to be increased with increasing grade (data not shown).Our findings and the literature reports emphasizing the tumor suppressor roles of let-7 family members and their downregulation in breast tumors compared to normal cells [11,42,43] not only prove that our meta-analysis tool is reliable but also increase the potential of let-7 family members and other miRNAs listed at the top of the meta-list as biomarkers for grade prediction. We also explored the miRNAs in meta-miRNA list associated with the gene expression grade index (GGI), which is believed to reflect and provide better proliferation and differentiation information than histological grade [44]. The differentially expressed miRNAs between GG low and GG high including let-7i, hsa-miR-379, hsa-miR-423 and hsa-miR-422a matched our meta-miRNA list and this was consistent with the findings of Rothe et al [41].

Next, we investigated the let-7 family targets in silico in order to further analyze their predictive potential regarding histological grade. The validated target gene lists were obtained for each miRNA (let-7c, let-7a, let-7f, let-7d, let-7e, let-7i, let-7g and let-7b). Further characterization of the genes was performed by pathway enrichment analysis. The genes were significantly enriched in pathways important for cancer development and progression such as Jak-STAT, MAPK, TGF-beta, mTOR signaling pathways, cell cycle and apoptosis (Fig 2). Since miRNAs destabilize mRNAs or repress translation by binding to the 3’UTRs of their targets, an increase in the expression level of a target gene is expected when the miRNA expression level decreases [8]. The decrease in the expression level of the let-7 family through dedifferentiation suggests the probability of an increase in the expression levels of target genes since one of the proposed mechanisms for miRNAs is mRNA destabilization. The fact that CEBPA, one of the common targets of these miRNAs, and the downstream genes E2F, IL6 and MYC have been shown to block differentiation and lead the cells to dedifferentiation supports this notion [45].

Many studies have shown the relation of miRNAs with the clinical status of breast cancer and reported their role in breast cancer pathogenesis, development and progression [20,41,46–49]. On the other hand, there are only a few studies focusing on the integrated analysis of a miRNA and the mRNA expression status in breast cancer [19,21,48]. In a study published in 2010, Guo et al. suggested the destabilization of target mRNAs (≥%84) rather than translational inhibition as the predominant reason for reduced protein output, pointing out mRNAs as main targets of miRNAs [8]. Data obtained from mRNA and protein expression level comparisons after introducing or deleting individual miRNAs also suggested mRNA destabilization to be the result of miRNA-mediated repression [50,51]. These data indicated the potential value of comparing meta-miRNA targets with grade-predictive mRNAs. Accordingly, we used the meta-analysis approach to independent mRNA microarray studies to determine the mRNAs that are predictive for breast cancer grade status. We expected the targets (mRNAs) of a miRNA with real predictive potential for grade status to be expressed differentially between grade 1, 2 and 3 samples. After obtaining the grade predictive meta-mRNAs by combining independent mRNA studies by our meta-analysis tool, we found 116 genes to be shared between the validated targets of let-7 family members and grade-predictive meta-mRNAs (Fig 4A, S2 Table). Pathway enrichment analysis was performed with the common 116 genes and most were found to be enriched in pathways related to cancer, TGF-beta signaling, the cell cycle, apoptosis, MAPK, mTOR and VEGF signaling. It is also important to mention that although the miRNA and mRNA microarray studies were from independent sources, they shared a number of mRNAs and miRNA targets that were found to be potential biomarkers for tumor grade. This consistency not only increases the possibility of the robustness of these 116 genes in tumor grade prediction but also stabilizes the biomarker roles of miRNAs targeting those mRNAs.

In conclusion, the meta-miRNAs that were obtained by the meta-analysis developed for this study could be verified experimentally in independent breast tumors and were comparable with previous studies related to breast cancer. We have demonstrated that the meta-analysis approach is reliable and robust and also that the let-7 family members of meta-miRNAs may be alternative and potential biomarkers for grade prediction. Meta-analysis of miRNA microarray datasets also has the potential to lead to more comprehensive measures of the existing differential miRNA expression data and can therefore provide miRNA and miRNA-target gene sets with high prognostic value.

## Supporting Information

S1 PRISMA Checklist PRISMA 2009 checklist. The page numbers corresponding to the items listed in the checklist were filled out according to the meta-analysis study presented.
(DOC)

S1 Table Meta-miRNA list. **miRNAs were noted according to their ranks in the list.** For each miRNA the mean of its rank in all studies was calculated with their p values (the mean of rank column) and this value became its *real rank* in the list within the real rank column, which was used instead of the significance value.
(XLSX)

S2 Table Common genes between the validated targets of let-7 family members and grade-predictive meta-mRNAs. (XLSX)
